# SIRT1: A Novel Target for the Treatment of Muscular Dystrophies

**DOI:** 10.1155/2016/6714686

**Published:** 2016-03-17

**Authors:** Atsushi Kuno, Yoshiyuki Horio

**Affiliations:** Department of Pharmacology, School of Medicine, Sapporo Medical University, Sapporo 060-8556, Japan

## Abstract

Muscular dystrophies are inherited myogenic disorders accompanied by progressive skeletal muscle weakness and degeneration. Duchenne muscular dystrophy (DMD) is the most common and severe form of muscular dystrophy and is caused by mutations in the gene that encodes the cytoskeletal protein dystrophin. The treatment for DMD is limited to glucocorticoids, which are associated with multiple side effects. Thus, the identification of novel therapeutic targets is urgently needed. SIRT1 is an NAD^+^-dependent histone/protein deacetylase that plays roles in diverse cellular processes, including stress resistance and cell survival. Studies have shown that SIRT1 activation provides beneficial effects in the dystrophin-deficient* mdx* mouse, a model of DMD. SIRT1 activation leads to the attenuation of oxidative stress and inflammation, a shift from the fast to slow myofiber phenotype, and the suppression of tissue fibrosis. Although further research is needed to clarify the molecular mechanisms underlying the protective role of SIRT1 in* mdx* mice, we propose SIRT1 as a novel therapeutic target for patients with muscular dystrophies.

## 1. Introduction

Muscular dystrophies are inherited diseases characterized by progressive muscle weakness and degeneration [1]. Mutations in genes encoding proteins that are essential for muscle cell stability, such as components of the dystrophin-glycoprotein complex, weaken the sarcolemmal membrane and cause various types of muscular dystrophy. Duchenne muscular dystrophy (DMD), the most common and severe form of muscular dystrophy, is an X-linked recessive genetic disorder caused by mutations in the gene that encodes dystrophin. Progressive muscle weakness usually begins as early as age 3, and affected patients typically lose their ability to walk before the age of 13. The life expectancy of patients with DMD typically ranges from 30 to 50 years of age. Although DMD causes heart and respiratory issues, mechanical ventilation prevents death from respiratory failure, and the leading cause of death is heart failure due to cardiomyopathy.

At present, the only approved therapeutics for patients with DMD are corticosteroids, which delay disease progression and improve muscle weakness. However, the use of corticosteroids is associated with several side effects, including weight gain and the development of gastric ulcers, metabolic diseases, and osteoporosis. Molecular approaches, including gene therapy, anti-sense-induced exon-skipping, and stop codon readthrough, which restore the production of functional dystrophin protein, are promising, and human clinical trials are currently in progress [2–4]. Because cardiac involvement in patients with DMD determines their prognosis, therapeutic interventions aimed at attenuating cardiomyopathy are also under investigation. Both angiotensin-converting enzyme inhibitors and *β*-blockers are reported to provide benefits in treating dystrophic cardiomyopathy [5]. In addition, a recent clinical trial demonstrated that the combination of the aldosterone blocker eplerenone and either angiotensin-converting enzyme inhibitors or angiotensin receptor blockers attenuates the progression of DMD-associated cardiac dysfunction [6]. However, these treatments are not sufficient to halt the progression of the disease.

Acetylation/deacetylation at lysine (Lys) residues is important posttranslational modification that regulates protein functions. Sirtuins are NAD^+^-dependent class III histone/protein deacetylases that are broadly conserved from bacteria to humans and have attracted attention because of their diverse physiological functions [7]. Among the seven mammalian sirtuins (SIRT1-7), SIRT1 is the most well-characterized and has been reported to regulate various cellular processes such as antioxidative stress, cell survival, cell cycling, cell differentiation, and metabolism by deacetylating target molecules [7]. SIRT1 is expressed in various tissues, including skeletal and cardiac muscles. Accumulating evidence indicates that SIRT1 plays roles in the muscle physiology and that the modulation of SIRT1 activity is potentially beneficial for treatment of muscle diseases [8, 9]. In this review, we will highlight the roles of SIRT1 in the muscle especially focusing on recent evidence showing the therapeutic potential against muscular dystrophies. We also discuss molecular mechanisms by which SIRT1 activation ameliorates muscle pathology in muscular dystrophies.

## 2. Beneficial Effects of SIRT1 Activation in an Animal Model of Muscular Dystrophy

Recently, we demonstrated the effect of treating dystrophin-deficient* mdx* mice with resveratrol [10], a potent activator of SIRT1 [11]. The treatment of 9-week-old* mdx* mice with 4 g resveratrol/kg chow (estimated to be approximately 500 mg/kg/day) for 32 weeks was found to significantly preserve muscle fiber mass in the biceps femoris. Resveratrol treatment also reduces interstitial fibrosis in the muscle of* mdx* mice. The SIRT1 expression level in the muscle of* mdx* mice is similar to that of controls and is not affected by resveratrol treatment. However, the histone H3 acetylation at Lys9/Lys14, deacetylation targets of SIRT1 [12], is increased in the muscle of* mdx* mice, and this increase can be suppressed by resveratrol treatment. These findings suggest that SIRT1 activity is attenuated in the muscle of* mdx* mice and that resveratrol treatment can reverse this effect. In addition, since resveratrol attenuates oxidative stress in the muscle tissues, the antioxidative function may underlie the protection by resveratrol. After our first report, several other groups also have shown the protective effect of resveratrol in the* mdx* mouse. Selsby et al. reported that overexpression of peroxisome proliferator-activated receptor gamma coactivator 1-alpha (PGC-1*α*), which is activated by SIRT1 [13–15], in the muscle, results in a fiber type shift, resistance to muscle injury, and muscle fatigue in the* mdx* mice [16]. Treating one-month-old* mdx* mice with resveratrol at 100 mg/kg/day for eight weeks mimics the beneficial effect of PGC-1*α* overexpression such as fatigue resistance. A report by Gordon et al. showed that infiltration of inflammatory cells in the skeletal muscle is attenuated by resveratrol treatment [17]. They also reported that resveratrol treatment improves muscle motor function in the* mdx* mouse [18]. In addition, Ljubicic et al. demonstrated that resveratrol induces oxidative fiber type conversion in the* mdx* mouse, which was associated with PGC-1*α* deacetylation and activation of AMP-activated protein kinase (AMPK), a serine/threonine kinase, by resveratrol in the muscle [19]. These reports indicate that resveratrol affords protection against muscle injury in the* mdx* mice via multiple mechanisms, which will be discussed later. Although resveratrol is reported to have multiple targets including AMPK [20] and phosphodiesterase [21] beside SIRT1, a recent report by Chalkiadaki et al. demonstrated that SIRT1 overexpression in the skeletal muscle of* mdx* mice attenuates muscle injury, as determined by serum creatine kinase activity and histological analyses and results in improved exercise capacity in a treadmill test [22]. These findings indicate that SIRT1 plays protective roles in the muscle of* mdx* mice and supports the notion that SIRT1 is involved in the resveratrol-induced protection of the muscle.

SIRT1 activation also plays protective roles against the cardiomyopathy associated with muscular dystrophy models [23]. We previously reported the effects of resveratrol in TO-2 hamsters which lack *δ*-sarcoglycan and develop severe dilated cardiomyopathy and heart failure [24]. *δ*-Sarcoglycan is a member of the dystrophin-associated glycoprotein complex and links the cytoskeleton to the extracellular matrix. Mutations in the gene encoding *δ*-sarcoglycan cause one of the less-common sarcoglycanopathies, limb-girdle muscular dystrophy type 2F. Defective *δ*-sarcoglycan also leads to the development of severe cardiomyopathy and subsequent heart failure [25]. The long-term treatment of TO-2 hamsters with resveratrol attenuates their cardiac hypertrophy and fibrosis, resulting in improved cardiac function. Notably, resveratrol treatment also extends the lifespan of TO-2 hamsters [24]. Since resveratrol treatment induces antioxidative superoxide dismutase 2 (SOD2) expression in the heart of TO-2 hamsters, suppression of oxidative stress may participate in protection afforded by resveratrol in this model. We also reported that the resveratrol treatment of dystrophin-deficient* mdx* mice also results in reduced cardiac hypertrophy/fibrosis and the preservation of cardiac function [26]. This effect was attributed to SIRT1-mediated degradation of p300 which plays critical roles in cardiac hypertrophy and tissue fibrosis. Since heart failure due to cardiomyopathy is currently the leading cause of death in patients with DMD, SIRT1 activation might provide a prognostic advantage in patients with muscular dystrophies.

## 3. Underlying Mechanisms by Which SIRT1 Activation Suppresses the Pathogenesis of Muscular Dystrophy

### 3.1. The Antioxidative Effect of SIRT1 Activation in Muscular Dystrophy

Oxidative stress has been implicated in the pathogenesis of muscle damage in DMD [27], and oxidative stress markers are elevated in the skeletal muscle tissues of patients with DMD [28, 29]. In the dystrophin-deficient* mdx* mouse, oxidative damage is detected in the muscle prior to the onset of muscle necrosis [30], suggesting that increased oxidative stress is involved in the development of muscle injury. Furthermore, treating 3-week-old mice with the antioxidant N-acetylcysteine (NAC) for 6 weeks reduces the percentage of centrally nucleated muscle fibers, demonstrating muscle fiber regeneration [31]. In addition, treatment of isolated and perfused muscles from* mdx* mice with NAC prevents contraction-induced muscle injury [31]. NAC is also reported to attenuate exercise-induced muscle injury in* mdx* mice, as determined by both histological analysis and creatine kinase release [32]. Notably, EUK-134, a catalytic mimetic of superoxide dismutase and catalase, was shown to reduce oxidative stress and muscle damage markers, including the percentage of muscle with central nuclei in the diaphragm of* mdx* mice [33]. In addition, we showed that the oxidative stress markers 8-hydroxyguanosine and nitrotyrosine are increased in the biceps femoris muscle of* mdx* mice and that these markers can be suppressed by resveratrol treatment [10]. Taken together, these findings suggest that targeting oxidative stress will be an effective strategy for treating muscular dystrophies and that SIRT1 activation protects muscles of* mdx* mice, at least in part, by suppressing oxidative stress.

Resveratrol's ability to suppress oxidative stress may involve its modulation of NADPH oxidase, a multiprotein complex that produces reactive oxygen species (ROS). NADPH oxidase was originally discovered in neutrophils [34] and is also expressed in skeletal and heart muscles. In the* mdx* mouse, NADPH oxidase has been implicated as a source of ROS in the muscle, although there is currently no evidence for this in DMD patients. The catalytic subunit of the NADPH oxidase complex is NOX, and seven NOX isoforms, encoded by separate genes, have been identified in mammals. Among them, NOX2, also known as gp91^phox^, is associated with p22^phox^ and requires p47^phox^, p67^phox^, and the small GTPase Rac for its activation. In contrast, NOX4 activity is unaffected by the absence of p47^phox^, p67^phox^, and Rac but requires the presence of p22^phox^. Previous reports, including ours, showed that* mdx* mouse muscle exhibits increased NOX activity [35] as well as the upregulated expression of NOX family members and other components of the complex [10, 36, 37]. Notably, the increased production of ROS in* mdx* mouse muscles was shown to be attenuated by the NOX inhibitor diphenylene iodonium or the NOX2-specific peptide inhibitor [35, 38]. In addition, the genetic deletion of p47^phox^ in the* mdx* mouse results in reduced ROS levels [38]. Our findings also showed that resveratrol treatment suppresses NOX4 and p47^phox^ upregulation in the muscle of* mdx* mice and that SIRT1 knockdown in cultured C2C12 myoblasts results in NOX4 and p47^phox^ protein upregulation [10]. These data suggest that SIRT1 activation attenuates oxidative stress in* mdx* mouse muscle by repressing the NOX4 and p47^phox^ expression.

The mechanism by which SIRT1 negatively regulates NOX4 and p47^phox^ expression is currently unknown. However, in human pulmonary artery smooth muscle cells and lung mesenchymal cells, NOX4 expression is induced by transforming growth factor- (TGF-) *β* receptor activation and depends on Smad2/3, key transcription factors in TGF-*β* signaling [39, 40]. Our studies showed that the TGF-*β* expression levels are higher in* mdx* mouse muscle than in control mouse muscle and are not altered by resveratrol treatment [10]. Since the SIRT1-mediated deacetylation of Smad3 is reported to attenuate its transcriptional activity [41, 42], SIRT1 may repress NOX4 expression via the suppression of Smad3 activity.

SIRT1 activation is also reported to counteract oxidative stress by indirectly inducing the expression of antioxidative molecules. Among the targets of SIRT1, the FoxO transcription factors (FoxOs) play important roles in oxidative stress resistance by upregulating the expression of ROS-detoxifying enzymes, including SOD2 and catalase [43]. SIRT1 is reported to deacetylate FoxO1 [44], FoxO3a [45], and FoxO4 [46, 47], which leads to their activation. We previously reported that SIRT1 is a nucleocytoplasmic shuttling protein [48] and that nuclear SIRT1 induces SOD2 expression, decreases ROS levels, and inhibits oxidative stress-induced cell death in C2C12 myoblasts [24]. SOD2 localizes to the mitochondria and plays a critical role in antioxidation by catalyzing the conversion of superoxide into hydrogen peroxide. Treating C2C12 cells with resveratrol results in SOD2 upregulation, an effect that can be suppressed by knockdown of either the FoxO transcription factors or SIRT1 [49, 50]. Furthermore, the finding that SOD2 knockdown prevents resveratrol-mediated cytoprotective effects [24] suggests that SOD2 is a primary downstream effector of the SIRT1 activator. In addition, the treatment of TO-2 hamsters with resveratrol results in SOD2 upregulation, which may also contribute to resveratrol's beneficial effects* in vivo* [24]. Notably, the SOD3 expression level is lower in the muscle of* mdx* mice than in that of control mice, whereas the SOD1 and SOD2 levels are unchanged. Unexpectedly, treating* mdx* mice with resveratrol was found to induce SOD1, but not SOD2 or SOD3 [10]. Although it remains unclear why resveratrol failed to upregulate SOD2 in the* mdx* mouse muscle, it is possible that the TGF-*β*-mediated repression of SOD2 [51] prevents its upregulation by resveratrol.

We also found that the levels of mRNAs that encode the components of gamma-glutamyl-cysteine ligase, the rate-limiting enzyme for glutathione biosynthesis (glutamyl-cysteine ligase modulator [Gclm] and glutamyl-cysteine ligase catalytic subunit [Gclc]) [52], are reduced in* mdx* mouse muscle [10]. Consistent with this finding, in patients with DMD, enhanced oxidative damage in the muscle is associated with glutathione depletion and reduced activities of gamma-glutamyl-cysteine ligase and glutathione peroxidase [29]. Both Gclm and Gclc are target genes of the nuclear factor erythroid 2-like factor 2 (Nrf2), a transcription factor that is activated in response to oxidative stress [52, 53]. In addition, the level of mRNA encoding Nqo1 (NAD(P)H-quinone oxidoreductase), another Nrf2-regulated gene [53], is reduced in* mdx* mice [10]. Thus, Nrf2 activation may be impaired in* mdx* mice, resulting in the downregulation of various antioxidants and enhanced oxidative stress in the muscles of these mice. Notably, however, resveratrol treatment does not affect Nrf2 target gene expression [10], indicating that SIRT1 plays a negligible role in the Nrf2 pathway in* mdx* mouse muscle.

### 3.2. Effects of SIRT1 Activation on Inflammation in Muscular Dystrophy

Myofiber necrosis promotes the invasion of inflammatory cells which release inflammatory cytokines that in turn enhance myofiber degeneration. Inflammatory cells are often found in the muscle of DMD patients [54] and may contribute to ROS production in the muscle tissue. Protein-DNA array analysis of the active transcription factors in* mdx* mouse muscle demonstrated that a number of transcription factors involved in inflammation are activated [55]. These include nuclear factor-kappa B (NF*κ*B), signal transducer and activator of transcription 3 (STAT3), and activator protein 1 (AP-1), each of which is known to be regulated by SIRT1. SIRT1 is reported to interact with the p65 subunit of NF*κ*B and deacetylate p65 at Lys310, which leads to suppressed NF*κ*B signaling [56]. SIRT1 also regulates the acetylation status of a cluster of C-terminal lysine sites (Lys685, 679, 707, and 709) in STAT3, and SIRT1 deacetylation at these sites inhibits STAT3 phosphorylation and transactivation [57]. Furthermore, SIRT1 decreases c-Fos/c-Jun acetylation and inhibits the transcriptional activity of AP-1 [58, 59]. Therefore, resveratrol treatment of* mdx* mice is expected to reduce muscle-associated inflammation. However, we found that resveratrol treatment failed to suppress the infiltration of leukocyte common antigen- (CD45-) positive inflammatory cells in the biceps femoris muscle of* mdx* mice and failed to suppress the increased levels of tumor necrosis factor- (TNF-) *α* and interleukin- (IL-) 1*β* mRNAs detected in the muscles of these mice [10]. In contrast, Gordon et al. reported that treating 5-week-old* mdx* mice with resveratrol at 100 mg/kg/day for 10 days reduces the infiltration of CD45-positive and F4/80-positive cells into the gastrocnemius muscle but has no effect on the muscle levels of TNF-*α* mRNA [17]. However, the effects of resveratrol on inflammation seem to be modest. Therefore, further research is needed to clarify the anti-inflammatory effect of SIRT1 in the dystrophic muscle, and a study using SIRT1 transgenic mice may provide its efficacy in the muscle inflammation in muscular dystrophy.

### 3.3. SIRT1-Regulated Fiber Type Shift in the Skeletal Muscle of* mdx* Mice

Skeletal muscle fibers are divided into two major types: slow (type I) and fast (type II) muscle fibers. Type I fibers primarily consist of type I myosin heavy chain (MHC) and slow troponin and generate ATP efficiently by oxidative phosphorylation, while type II fibers primarily consist of type IIb MHC and fast troponin and generate ATP by glycolysis. Intermediate fiber types also correlate with the MHC isoforms they express, which range from the fastest to the slowest isoform in the order MHC IIb, IIx, IIa, and I. The slower oxidative muscle fibers have been shown to be less susceptible to developing the dystrophic pathology [60].

Differences in the muscle fiber composition in* mdx* and control mice have been reported. The percentage of type I MHC-positive fibers in the slower, more oxidative soleus muscle is significantly lower in the* mdx* versus control mice. In addition, the percentage of relatively oxidative MHC IIa-positive fibers in the fast, more glycolytic extensor digitorum longus (EDL) and tibialis anterior (TA) muscles is also reduced in* mdx* mice [19]. Consistent with these findings, the other reports demonstrated that the muscles of* mdx* mice contain a higher percentage of type II glycolytic fibers than those of control mice [16, 37]. Taken together, these data suggest that the dystrophic muscles in the* mdx* mice may be associated with a slow to fast fiber switch and that the conversion of these fibers to a slower and more oxidative phenotype could be beneficial in recovering the function of dystrophic muscle.

PGC-1*α* is highly expressed in the skeletal muscle and plays a role in muscle adaptation in response to physiological and pathophysiological conditions. It is reported that PGC-1*α* expression is induced by exercise in the skeletal muscle [61, 62]. Furthermore, PGC-1*α* overexpression in the skeletal muscle of normal mice induces the muscle fiber type switching from type II to type I [63], indicating a key role of PGC-1*α* in the fast-to-oxidative fiber type shift in the skeletal muscle. In the* mdx* mouse, PGC-1*α* overexpression in skeletal muscle also results in a fast to slow fiber type shift, a restoration of* in vitro* muscle function, and attenuation of muscle injury [16]. The acetyltransferase GCN5 increases the PGC-1*α* acetylation, resulting in reduced PGC-1*α* activity [64]. Conversely, the deacetylation of PGC-1*α* by SIRT1 increases its activity [13–15]. Notably, SIRT1 overexpression in skeletal muscle induces a fast to slow fiber type switch in healthy mice [22], and resveratrol treatment induces the development of oxidative type muscle fibers in high fat diet-fed mice [65]. These findings suggest that increased SIRT1 activity has the potential to induce a fast to slow fiber shift in the muscle of* mdx* mice. Consistent with this notion, Ljubicic et al. [19] demonstrated that the resveratrol treatment of* mdx* mice restores type I MHC-positive fiber development in the soleus muscle and partially restores type IIa MHC-positive fiber development in the EDL and TA muscles. They showed that resveratrol increases SIRT1 expression and activity and reduces PGC-1*α* acetylation level in dystrophic muscle [19]. We also reported that resveratrol treatment upregulates the mRNAs that encode type I MHC and slow troponin in the biceps femoris of the* mdx* mouse [10].

Since slow oxidative fibers are rich in mitochondria, the fast to slow fiber type shift may be accompanied by an increase in mitochondrial content. Consistent with this prediction, PGC-1*α* has been implicated in mitochondrial biogenesis [66]. Furthermore, treating high fat diet-fed mice with resveratrol induces mitochondrial DNA and mitochondria-related gene upregulation in the gastrocnemius muscle [65]. The transgenic overexpression of SIRT1 also results in increased mitochondrial content and activity in muscle [22]. The finding that PGC-1*α* deacetylation is promoted by resveratrol or SIRT1 overexpression [22, 65] suggests that PGC-1*α* may mediate SIRT1 activation-induced mitochondrial biogenesis. In agreement with these findings, the resveratrol-induced fast to slow fiber switch in* mdx* mice was found to be associated with the upregulation of cytochrome c oxidase subunit IV (COX4) [19].

### 3.4. SIRT1 Antifibrotic Action

Enhanced organ fibrosis compromises tissue function and leads to organ dysfunction in a variety of diseases. Tissue fibrosis is also a prominent pathological feature of the dystrophic skeletal muscle in DMD patients. The recent finding that endomysial fibrosis is associated with poor motor outcome in patients with DMD [67] suggests that the suppression of muscle fibrosis may be a beneficial treatment strategy for patients with muscular dystrophy. Notably, we found that resveratrol treatment significantly suppresses interstitial fibrosis in the biceps femoris of* mdx* mice [10]. Thus, the antifibrotic effect associated with SIRT1 activation may be advantageous in treating muscular dystrophies.

TGF-*β* receptor activation and downstream signaling contribute to the pathogenesis of a variety of fibrotic disorders [68]. Notably, TGF-*β*1 expression levels were found to be correlated with fibrosis in muscle biopsies from patients with DMD [69], and TGF-*β* signaling-related genes are upregulated in the muscle from symptomatic DMD patients [70]. As described above, TGF-*β* expression is reported to be upregulated in* mdx* mouse muscles [10, 71, 72]. The TGF-*β*-mediated differentiation of myofibroblasts results in the secretion of extracellular matrix components, such as collagens and fibronectin, which promote fibrotic changes in the tissue [68]. Since resveratrol-mediated fibrosis suppression is associated with the downregulation of *α*-smooth muscle actin, a myofibroblast marker, the inhibition of myofibroblast differentiation is likely to be responsible for the antifibrotic effect of resveratrol [10]. The finding that resveratrol does not suppress TGF-*β* upregulation in* mdx* mouse muscle [10] suggests that signaling components downstream of the TGF-*β* receptor may be resveratrol targets. Although the SIRT1 target in* mdx* mice has not yet been identified, there are several possible candidates. First, as described above, we found that resveratrol suppresses the NOX4 upregulation in* mdx* mouse muscle. Since NOX4 is a critical mediator of myofibroblast differentiation and tissue fibrosis [40, 73, 74], NOX4 repression may contribute to the antifibrotic effect of resveratrol. Second, SIRT1 may modulate the activity of the Smad transcription factors, key TGF-*β* signaling components that are involved in myofibroblast differentiation. The activity of Smads is regulated by lysine acetylation/deacetylation, which plays a critical role in tissue fibrosis [75]. The transcription coactivator p300 is reported to bind to and acetylate Smad2 and Smad3, which increases their transcriptional activity [76, 77]. In contrast, the deacetylation of Smad3 and Smad4 by SIRT1 is reported to reduce their activity [41, 78]. Furthermore, we recently reported that SIRT1 activation decreases the p300 protein level via deacetylation and subsequent polyubiquitination-mediated proteasomal degradation [26]. Thus, the downregulation of p300 by SIRT1 may contribute to the suppression of Smad transcription factors. Third, it appears that TGF-*β* signaling is inhibited by AMPK [79, 80]. Since AMPK is positively regulated by SIRT1 [81], SIRT1 may influence TGF-*β* signaling via AMPK activation.

### 3.5. Other Possible Mechanisms Modulated by SIRT1

In skeletal muscle, AMPK promotes mitochondrial biogenesis [82], fatty acid oxidation [83, 84], and glucose uptake [85]. Recent studies have demonstrated the crosstalk among SIRT1, AMPK, and PGC-1*α* to regulate muscle physiology. Jäer et al. found that pharmacological AMPK activation by using 5-aminoimidazole-4-carboxamide riboside (AICAR) and metformin increased mitochondria-related genes including cytochrome c and uncoupler protein 2, which is canceled in PGC-1*α*-deficient myotubes [82]. In this report, the authors demonstrated that phosphorylation of PGC-1*α* at Thr177 and Ser538 is required for action of AMPK. Liver kinase B1 (LKB1) is an upstream kinase that phosphorylates Thr172 in the AMPK*α* catalytic subunit, which is required for its activation. SIRT1-mediated LKB1 deacetylation at Lys48 is reported to increase the LKB1 activity, which leads to AMPK activation [81]. In agreement with this finding, treating high fat diet-fed mice with resveratrol at 25–30 mg/kg/day was found to induce AMPK*α* phosphorylation in the skeletal muscle of wild-type, but not SIRT1 knockout mice [86]. In contrast, Cantó et al. showed that pharmacological and exercise-induced activation of AMPK promotes deacetylation of PGC-1*α* in myotubes and skeletal muscles, which is mediated via SIRT1 [87]. SIRT1 activation by AMPK seems to attribute to an increase in the intracellular NAD^+^/NADH ratio [87, 88]. Furthermore, the same group demonstrated that PGC-1*α* deacetylation and activation in the muscle induced by fasting or exercise were not observed in AMPK*γ*3 knockout mice [89]. Although further work is required to determine the relationship between SIRT1 and AMPK, SIRT1/AMPK/PGC-1*α* signaling is the network that plays a physiological role in the skeletal muscle.

Treating* mdx* mice with AICAR was found to induce an increase in type IIa MHC-positive myofibers and COX4 levels in the EDL muscle [90]. Although this AICAR-induced shift in muscle fiber phenotype is accompanied by reduced membrane fragility, as determined in an* ex vivo* analysis [90], the same dose of AICAR fails to improve exercise performance in* mdx* mice [91]. In our study, we did not examine the AMPK activity in the muscle of* mdx* mice treated with 500 mg/kg/day of resveratrol. Notably, treating* mdx* mice with resveratrol at 100 mg/kg/day has no effect on phospho-AMPK*α* levels in the EDL muscle [19]. In addition, an earlier report showed that while a low dose of resveratrol (25–30 mg/kg/day) has no effect on phospho-AMPK*α* levels, a higher dose (215–235 mg/kg/day) can elevate the phospho-AMPK*α* even in SIRT1 knockout mice [86]. Further analyses are needed to clarify AMPK's involvement in the SIRT1-mediated protective effects in dystrophic muscles.

Autophagy is a highly conserved process in which cytosolic proteins and organelles are sequestered into an autophagosome, then delivered into a lysosome, and degraded. Recent studies have uncovered a physiological role of autophagy in maintaining skeletal muscle [92–94]. Autophagic activity is impaired in the skeletal muscles of* Col6al* ^−*/*−^ muscular dystrophic mice, and genetic and pharmacological interventions that restore autophagy ameliorate the dystrophic phenotype of these mice [95]. In addition, autophagy is reported to be impaired in the skeletal muscle of* mdx* mice and human DMD patients [38, 96–98]. Cells use autophagy to balance their energy sources in response to nutrient stress, and the energy-sensing protein SIRT1 has been shown to positively regulate autophagy via diverse pathways. SIRT1 stimulates autophagy through the deacetylation of autophagy components, including Atg5, Atg7, and Atg8 (also known as microtubule-associated protein light chain 3 (LC3)) [99, 100]. SIRT1 also enhances the transcription of autophagy-related genes. For example, SIRT1 deacetylates and activates FoxO3a [45], a transcription factor that regulates autophagy in skeletal muscle [101, 102]. In cardiac myocytes, SIRT1 is reported to activate FoxO1, leading to the upregulation of Rab7, a small GTP-binding protein that mediates late autophagosome-lysosome fusion [103]. Taken together, these findings suggest that the SIRT1-mediated improvement in muscle function in* mdx* mice may be due in part to the restoration of autophagy.

## 4. Future Prospects

To develop new therapeutics for a human disease, it is critical to select the proper animal model. The most extensively used model for DMD is the* mdx* mouse, which lacks dystrophin. Although this mouse has been used to investigate the disease pathogenesis and to test the efficacy of various interventions, it retains a normal lifespan, and the muscle damage observed in this mouse is much less severe than that in the human disease. The relatively mild phenotype is probably due to the compensatory upregulation of utrophin, a cytoskeletal protein whose structure is similar to that of dystrophin. Notably, compared to* mdx* mice,* mdx*-utrophin double knockout mice [104] exhibit a more severe disease phenotype and a shorter lifespan. In addition, canine models of DMD, including the golden retriever muscular dystrophy model, also have a more severe phenotype than that of the* mdx* mouse. The evaluation of SIRT1 activators in animal models whose severity and pathogenesis more closely reflect the human disease may better predict their efficacies.

In our study, resveratrol treatment significantly attenuated the muscle pathology associated with the* mdx* mouse. However, the elevation in serum creatine kinase, a marker of muscle injury, was not attenuated by resveratrol treatment [10]. Since Chalkiadaki et al. reported that skeletal muscle-specific SIRT1 overexpression blocks the increased serum creatine kinase levels in the* mdx* mouse [22], we cannot exclude the possibility that resveratrol's potency for activating SIRT1 is not sufficient to suppress muscle injury. Thus, a stronger SIRT1 activator may be required for treating patients with DMD. Synthetic SIRT1 activators with an improved selectivity for SIRT1 have been reported [105]. Among them, SRT2104 is reported to be well tolerated in healthy adults [106] and elderly volunteers [107]. The administration of SRT2140 (2 g/day) was found to be tolerable and safe in healthy smokers; however, there were no effects on serum lipid profiles, plasma fibrinolytic factors, and markers of platelet and monocyte activation [108]. In contrast, administration of the same compound at the same dose reduced the endotoxin-induced cytokine release and coagulation activation in humans [109]. Recently, Krueger et al. reported the effects of SRT2104 in patients with moderate to severe psoriasis [110]. Although the sample size was small, SRT2104 (250~1000 mg/day) was found to improve the histological findings and to attenuate the expression levels of TNF-*α*/IL-17 pathway-related genes, which are known to play roles in psoriatic skin pathogenesis. These findings suggest that SRT2104 may exert anti-inflammatory effects in patients with DMD.

## 5. Conclusions

**Figure 1:**
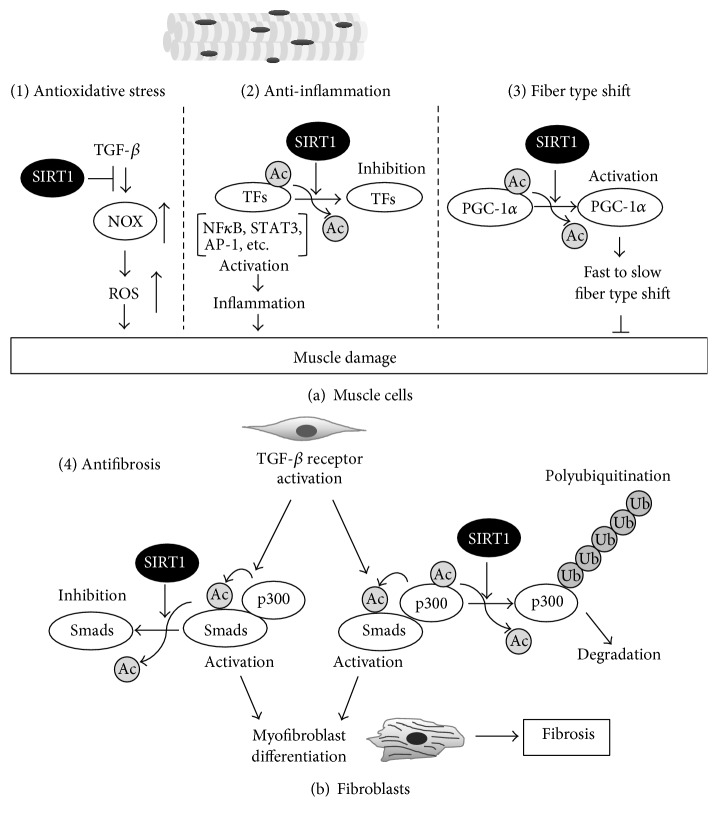
Mechanisms of protection by SIRT1 activation against muscular dystrophy in muscle cells (a) and fibroblasts (b). (a) In muscle cells, activation of SIRT1 suppresses oxidative stress by inhibiting transforming growth factor-*β*- (TGF-*β*-) induced upregulation of NADPH oxidase (NOX), which produces reactive oxygen species (ROS) (1). SIRT1 is reported to inhibit transcription factors (TFs) related to inflammatory responses via deacetylation, which leads to suppression of muscle inflammation (2). Activation of peroxisome proliferator-activated receptor gamma coactivator 1-alpha (PGC-1*α*) by SIRT1-mediated deacetylation promotes fast to slow fiber shift (3). (b) Tissue fibrosis is attenuated by SIRT1 via two mechanisms in fibroblasts. One is SIRT1-mediated deacetylation and inhibition of Smad transcription factors. The other is p300 deacetylation by SIRT1 and subsequent p300 protein degradation via the ubiquitin-proteasome pathway. NF*κ*B: nuclear factor-kappa B; STAT3: signal transducer and activator of transcription 3; AP-1: activator protein 1; Ac: acetyl group; Ub: ubiquitin.

Studies have shown that SIRT1 activation provides beneficial effects in the dystrophin-deficient* mdx* mouse. These effects are attributed to the multiple biological functions of SIRT1, including the attenuation of oxidative stress and inflammation, promotion of fast to slow myofiber shift, and suppression of tissue fibrosis (Figure 1). Although further research is needed to clarify the molecular mechanisms by which SIRT1 protects muscle function in* mdx* mice and to translate the outcome of preclinical studies into clinical applications, we propose that SIRT1 activation represents a novel therapeutic strategy for patients with muscular dystrophies.

## Abbreviations

AICAR: 5-Aminoimidazole-4-carboxamide riboside
AMPK: AMP-activated protein kinase
AP-1: Activator protein 1
DMD: Duchenne muscular dystrophy
EDL: Extensor digitorum longus
FoxO: Forkhead box O
Gclc: Glutamyl-cysteine ligase catalytic subunit
Gclm: Glutamyl-cysteine ligase modulator
IL: Interleukin
LC3: Microtubule-associated protein light chain 3
LKB1: Liver kinase B1
MHC: Myosin heavy chain
NAC: N-Acetylcysteine
NAD: Nicotinamide adenine dinucleotide
NADPH: Nicotinamide adenine dinucleotide phosphate
NF*κ*B: Nuclear factor-kappa B
NOX: Nicotinamide adenine dinucleotide phosphate oxidase
Nrf2: Nuclear factor erythroid 2-like factor 2
Nqo1: NAD(P)H-quinone oxidoreductase
PGC-1*α*: Peroxisome proliferator-activated receptor gamma coactivator 1-alpha
ROS: Reactive oxygen species
SOD: Superoxide dismutase
STAT3: Signal transducer and activator of transcription 3
TA: Tibialis anterior
TGF-*β*: Transforming growth factor-*β*
TNF-*α*: Tumor necrosis factor-*α*.
